# Supplementary material for: Inhomogeneous spatial distribution of the magnetic transition in an iron-rhodium thin film
Source: Nat Commun. 2017 Jun 7;8:15703. doi: 10.1038/ncomms15703 (PMC5467229; doi:10.1038/ncomms15703)
Supplement: Supplementary Information — Supplementary Figures, Supplementary Notes and Supplementary References [file ncomms15703-s1.pdf]

Supplementary Note 1
Description of the sample and thin lamella preparation

A 50nm FeRh layer was epitaxially grown on a MgO (001) substrate by DC sputtering using a co-deposition process from two pure Fe and Rh targets. DC currents on the magnetrons were set to get the proper composition that will give the magnetic transition at room temperature. The layer was deposited at 550°C and annealed for 6 hours at 800°C. The antiferromagnetic/ferromagnetic (AF/F) transition has then been checked by Vibrating Sample Magnetometry (VSM) on the whole sample.

For Electron Holography (EH) experiments, the thickness crossed by the electrons needs to be uniform to ensure a quantitative treatment. The Focused Ion Beam (FIB) process has therefore been chosen to prepare the thin lamella. The FeRh layer was protected by a 150nm thick ink layer and a 250nm thick Pt layer to avoid damages and charge accumulation during the thinning process, as MgO is an insulator. The lamella was then extracted and thinned down to 100nm to get electron transparency. The final step is a low energy one to minimize irradiation damages and amorphization of the surfaces. Supplementary Fig 1a presents a low magnification TEM image of the whole FIB lamella, the area chosen for the EH study is marked by a yellow rectangle. The image displayed in Supplementary Fig 1b has been obtained by Scanning Electron Microscopy (SEM) tilting the sample at 90°. The SEM image was used to perform more accurately measurement of the lamella width crossed by the electron beam.

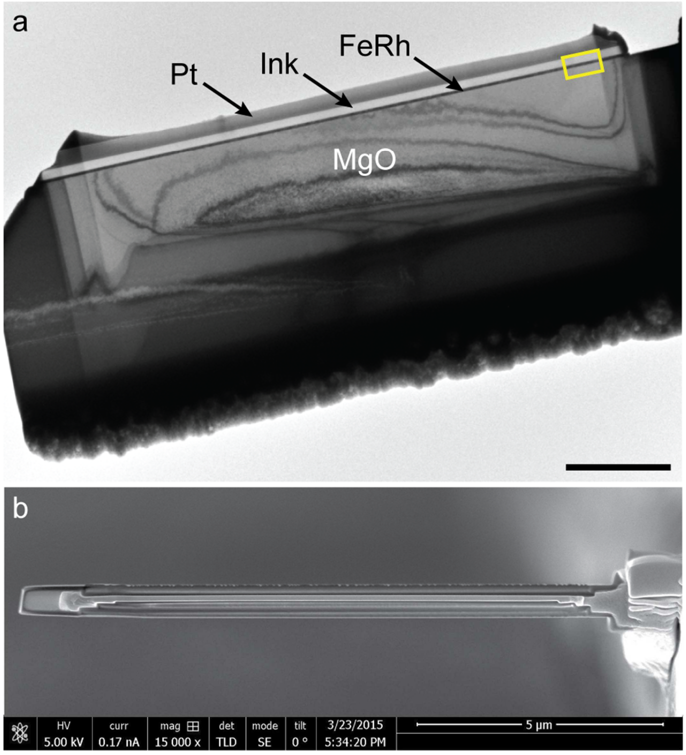

Supplementary Figure 1 | FeRh lamella prepared by FIB and used for *in situ* electron holography experiments . (**a**) Conventional TEM image of the thin lamella in cross-sectional view. The scale is 2 µm. The ink and Pt protecting layers are indicated on the top of the FeRh/MgO sample. The yellow rectangle at right corresponds to the area studied by EH. (**b**) Top view SEM image of the lamella to measure its thickness (≈ 90 nm) crossed by the electron beam. No bending has been observed. The scale bar represents 5 µm.

Supplementary Note 2
Transmission Electron Microscope and heating-cooling sample holder

A Hitachi HF 3300C microscope operating at 300kV was used for the EH experiments. This microscope is dedicated to electron interferometry and *in situ* TEM experiments. A cold field emission gun provides high spatial and temporal coherence for interferometric studies. The high mechanic and electronic stability favours a better signal over noise ratio. Dedicated Lorentz modes combined with the B-core corrector were developed to achieve a 0.5nm spatial resolution in a field-free magnetic environment (less than 10 Oe). The Lorentz mode used for this experiment is the TL11 mode: the objective lens is switched off so that its magnetic field does not interact with the magnetic induction of the FeRh layer. The objective lens is replaced by the first lens of the corrector, the TL11 lens, located below. However the objective lens can then be turned on for applying a magnetic field (up to 1 T) on the sample and for controlling by this way the magnetic state of the sample.

All the holograms were recorded in a 2 biprism configuration[1] to avoid artefacts linked to Fresnel fringes and to set separately the interference area size and the fringe spacing. The fringe spacing was set to 0.9nm in this study.

The sample holder is a single tilt Gatan HC3500 holder allowing a temperature control from -150°C to +250°C. A liquid nitrogen tank is located on the sample holder, connected to the specimen carrier by a metallic cupper braid. An electric resistance is used to heat locally close to the sample and a thermocouple provides the real-time temperature. The entire system is controlled by PID (Proportional Integral Derivative) using software directly manipulated by the user.

Supplementary Note 3
Methods and hologram series

The temperatures applied to the sample to follow the AF/F transition are between -120°C and + 125°C. The study by EH began with stabilizing a temperature of + 125°C to get the sample into the ferromagnetic state. This state was *in situ* confirmed from the experimental phase image using a live processing of the hologram.

In order to avoid strong dynamical diffraction effects, the layer was slightly tilted away from the [-110] zone axis. It is then *in situ* magnetically saturated parallel to the [110] direction of FeRh (noted *x*) prior to the observation at the remnant state: the maximum magnetic field of the objective lens (about 1 T) was applied after tilting the sample by +60° along the growth direction. The component of the applied field in the sample plane is therefore 1T.sin(+60°) = 0.866T. Then the objective lens is turned off again before getting the sample back to zero tilt. A first set of 20 holograms was acquired during the decrease in temperature from +125°C to -120°C before recording a second set of 35 holograms during the rise in temperature up to + 125°C. A third set of 22 holograms was recorded by decreasing temperature down to -120°C but after *in situ* magnetic saturation at + 125°C in the direction opposite to the previous saturation, i.e. with tilting the sample at -60°: : in this way we ensure the reproducibility of the transition and the influence of the initial magnetic state

All the holograms were acquired over the same area with a 5s exposure time after temperature stabilization to perform a static study of the transition. For each hologram obtained on the area of interest, a reference hologram was recorded immediately after in vacuum. We checked between the beginning and the end of the experiment that the FeRh layer has not been modified by the irradiation of the electron beam by comparing the amplitude images, but also the magnetic phase images obtained in the same ferromagnetic state at 125°C.

Supplementary Note 4
Principle of electron Holography

Off-axis electron holography[2,3] (EH) is a powerful interferometric method that can be carried out in a Transmission Electron Microscope (TEM). A beam of highly coherent electrons that has interacted with an object and the surrounding electromagnetic fields (object wave) is interfered with a part of the beam (reference wave) that has not interacted with any field (Supplementary Fig 2). The resulting interference pattern is called a hologram and contains all the necessary information on the electron phase shift created by the local fields. The phase shift analysis allows obtaining quantitative maps of the local electric and magnetic fields.

To understand the unique capabilities of EH, two electron waves have thus to be considered:

- The reference wave can be written:

$$\psi_0(\mathbf{R}) = A_0 \exp i(\mathbf{k} \cdot \mathbf{R}) \qquad (1)$$

where $\mathbf{R}$ represents the position vector, A_0 the amplitude of the wave and $\mathbf{k}$ its wave vector. The phase at the origin is taken to be equal to zero by convention.

- The object beam that has been phase shifted after interaction with the sample can be written:

$$\psi(\mathbf{R}) = A(\mathbf{r}) \exp i((\mathbf{k} \cdot \mathbf{R}) + \phi(\mathbf{r})) \qquad (2)$$

where $\mathbf{r}$ is the position vector in the plane of the sample (perpendicular to the beam), $A(\mathbf{r})$ the amplitude of the wave modified by the sample and $\phi(\mathbf{r})$ the phase shift of the beam due to the sample.

In normal conditions of imaging, detection systems (CCD cameras) only record the intensity *I* of the beam i.e. $I = |\psi(\mathbf{R}) \cdot \psi*(\mathbf{R})|^2 = A(\mathbf{r})^2$. All the information carried by $\phi(\mathbf{r})$ is thus lost. On the contrary, the intensity of the hologram which results from the superposition of the two waves keeps the phase shift term:

$$I = |A_0|^2 + |A(\mathbf{r})|^2 + 2A_0 A(\mathbf{r}) \cos(\mathbf{k} \cdot \mathbf{R} + \phi(\mathbf{r})) \qquad (3)$$

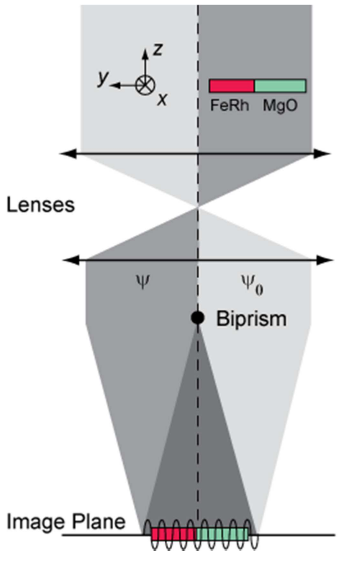

Supplementary Figure 2 | Off-axis electron holography set-up. The reference wave and the object wave are represented by two different levels of grey above the lenses. The superimposition of these below the biprism creates an interference pattern on the image plane.

This phase shift term contains the contribution of the electromagnetic fields through the Aharonov-Bohm (A - B) effect:[4,5]

$$\phi(\mathbf{r}) = C_{\mathrm{E}} \int V(\mathbf{r},z)dz - \frac{e}{\hbar}\int A_z(\mathbf{r},z)dz = \phi_{\mathrm{E}}(\mathbf{r}) + \phi_{\mathrm{M}}(\mathbf{r}) \quad (4)$$

where *z* is the direction parallel to the electron beam, C_E a constant related to the energy of the beam (7.29 10^6 $V^{-1}.m^{-1}$ at 200 kV,6.52 10^6 $V^{-1}.m^{-1}$ at 300 kV), *V* the electrostatic potential, *e* the electron charge, $\hbar$ the reduced Planck constant and A_z the *z* component of the vector potential (magnetic potential).

The A-B effect is thus separated into two distinct parts: the first term of the supplementary equation 4 originates from the electrostatic contribution (noted $\phi_{\mathrm{E}}(\mathbf{r})$) and the second the magnetic contribution (noted $\phi_{\mathrm{M}}(\mathbf{r})$). This expression provides the direct relation between the electron beam phase shift and the electromagnetic potential associated to the electrostatic and magnetic fields integrated along the beam path.

The total phase shift can be written as a function of the corresponding fields (**E** electric field and **B** magnetic induction):

$$\phi_{\mathrm{E}}(\mathbf{r}) = C_{\mathrm{E}} \iint \mathbf{E}(\mathbf{r},z)dr\,dz \text{ and } \phi_{\mathrm{M}}(\mathbf{r}) = -\frac{e}{\hbar}\iint \mathbf{B}_{\perp}(\mathbf{r},z)drdz \quad (5)$$

These electromagnetic contributions have a very high sensitivity.[6] To study quantitatively the magnetic properties of the sample, both contributions have to be separated. We have then recorded holograms at +125°C after having saturated the magnetization parallel to the FeRh layer in both opposite directions: the electrostatic contribution ϕ_{E} to the phase shift remains similar in the two holograms while the magnetic contribution ϕ_{M} changes in sign. The half sum of the corresponding phase images provides ϕ_{E}, which is subtracted from all the phase images to retain only ϕ_{M}. We checked that ϕ_{E} remains the same during the whole experiment by recording holograms in both saturated magnetic states at the beginning of each series of holograms and by repeating the same procedure to extract it.

It is very important to note that the measured magnetic phase shift corresponds to a projection and an integration of all in-plane magnetic field contributions along the electron path. In addition, numerical simulation are generally unavoidable to take into account the two dimensional projection of a three dimensional field.

Supplementary Note 5
Data treatment

Phase and amplitude images were extracted from the holograms by using homemade software following this procedure: a fast Fourier transform (FFT) of the hologram is calculated, a digital mask is then applied to the FFT on one sideband before being centered and the Inverse Fourier Transform (FFT^{-1}) allows calculating two images: the amplitude of the interference fringes and the relative phase shift of these fringes (phase image) which represents the displacement and periodicity variations compared to a reference area of the phase image. The size of the digital mask used in the FFT was chosen to obtain a spatial resolution of 2.5 nm.

The data processing has to take into account the additional phase distortions due to the microscope itself and to the detection system. This is achieved by calculating a reference phase image from a reference hologram recorded without any object and subtracting this reference phase image to the object one.

Amplitude images, which remain the same regardless of the applied temperature, were automatically realigned by a cross-correlation method. This realignment was then applied on the phase images.

To illustrate the procedure for induction quantification, we will take the magnetic phase image obtained +125°C (Supplementary Fig 3a) as an example. In the following, the [110] and [001] directions of FeRh were chosen as *x*-axis and *y*-axis respectively. The direction parallel to the electron

beam stands for the *z*-axis. The shape anisotropy lays the magnetization along the [110] direction within the FeRh layer.

The aim of this quantification is to extract the *x* component of the magnetization, noted M_x, and to study its evolution as a function of the temperature. The extraction of quantitative data was performed according to the following steps:

• A portion of the FeRh layer is selected by the user by means of a rectangle whose sides are parallel to the *x* and *y* directions (Supplementary Fig 3a).

• Within the rectangle, the profile extracted along the *y* direction (Supplementary Fig 3b) is averaged along the *x* direction over the entire length of the rectangle.

• A 1st order fit (linear) is calculated based on the averaged profile (Supplementary Fig 3c) to precisely extract the average slope of the curve along the *y* direction. This slope corresponds to:

$$\frac{\partial \phi_{\mathrm{M}}(\mathbf{r})}{\partial y} = \frac{e}{\hbar} \int B_x(\mathbf{r}, z)\, dz \qquad (6)$$

• Measuring the lamella width *w* crossed by the electron beam and assuming that the magnetization is equivalent to the induction inside the FeRh layer, it is then possible to obtain M_x:

$$\mu_0 M_x = \frac{\int B_x(\mathbf{r}, z)\, dz}{w} == \frac{\hbar}{ew} \frac{\partial \phi_{\mathrm{M}}(\mathbf{r})}{\partial y} \qquad (7)$$

• Repeating this analysis as a function of the applied temperature *T*, we then can plot $M_x = f(T)$ on a very localized area (Supplementary Fig 4). The resulting graph is therefore strictly equivalent to the classical *M*(*T*) curve obtained by magnetometry (VSM,…).

.

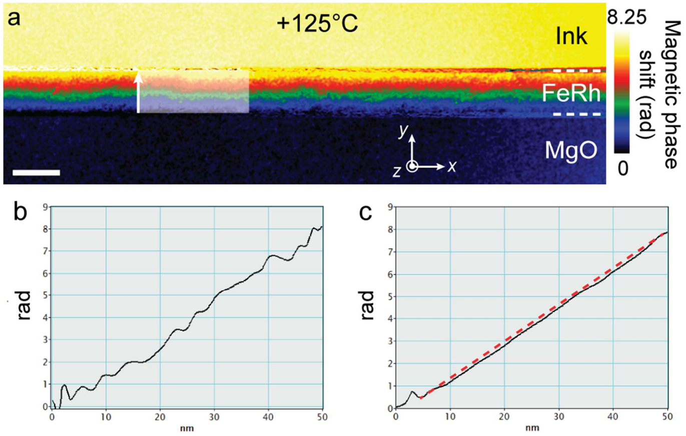

Supplementary Figure 3 | Principle of the data treatment. (**a**) Magnetic phase image obtained by EH on the FeRh layer to + 125 ° C with the system of axes. The scale bar is 50 nm. The arrow represents the direction of the extracted profile and the white rectangle defines the width used for the averaging. (**b**) Profile of the phase shift obtained along the white arrow on (**a**) parallel to the y direction inside the layer of FeRh. (**c**) In black, profile averaged along the x direction on the length of the white rectangle and in red, linear fit of the profile to extract the slope of the phase shift. This slope is directly proportional to the integrated induction.

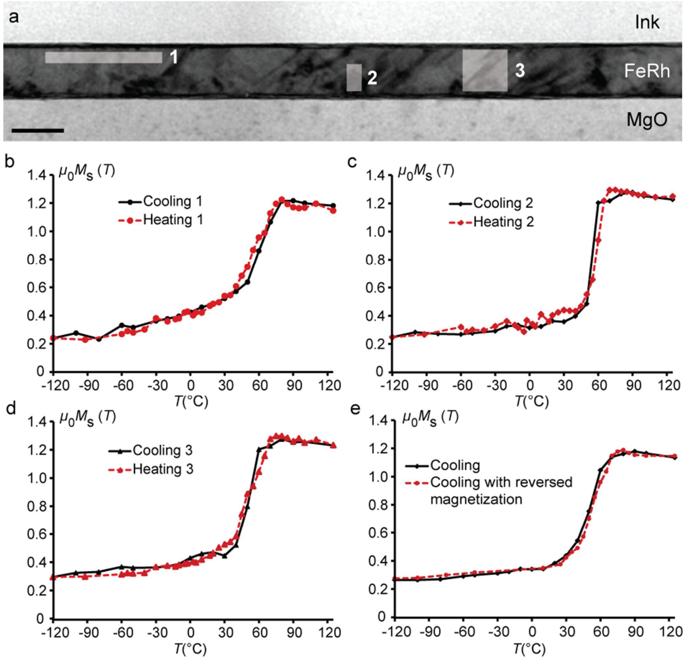

Supplementary Figure 4 | Magnetization vs temperature curves extracted at different locations. (**a**) Amplitude image of the studied area. The scale bar is 50 nm. (**b**) to (**d**) Magnetization as a function of the temperature for different investigated areas. (**e**) Magnetization as a function of the temperature (only for decreasing temperature) obtained on the whole field of view for the initial and the reversed magnetic saturation states. The two half cycles are very close and no effect related to the initial direction of magnetization is demonstrated.

A note should be made on the exploitable area: the difficulty of accurately and permanently set the focus in Lorentz mode throughout the duration of the experiment, and the multiple image realignments to extract the magnetic contribution and allow exploitation of results depending on the temperature, imply that it is not possible to operate the signal over the entire thickness of the FeRh layer. Small cumulative errors during realignments, combined with a spatial resolution of about 2 nm of the phase images broaden strong local variations in the signal at the ink/FeRh and FeRh/MgO interfaces. Thus the slope of the magnetic phase in the vicinity of the interfaces cannot be analysed without being strongly affected by these artefacts (this region is around 3nm thick in the FeRh part). This reduces the thickness of usable layer from 50nm to about 44 nm.

Supplementary Note 6
Micromagnetic simulations

Micromagnetic simulations were performed using the OOMMF 3D package (http://math.nist.gov/oommf). The universe used for calculation has dimensions of 660 nm along the *x* direction, 330 nm along the *y* direction and 330 nm in the *z* direction (direction parallel to the electron beam) with a size cell of 2x2x2 nm. The simulated FeRh layer has a thickness of 50 nm along the growth direction (*y* direction) and a width (thickness crossed by the electron beam) of 90 nm from measurements done during the FIB process. This width is divided into 3 layers: a layer of 80 nm thick composed of ordered FeRh presenting the AFM/FM transition separates two others layers of 5 nm thick perpendicular to the electron beam corresponding to the amorphized surface of the lamella by the FIB preparation (Supplementary Fig 5). 3 temperatures (25°C, 50°C and 60°C) have been investigated. The damaged layers were kept in a ferromagnetic state for all applied temperature with a magnetization equal to 1.19 T. This magnetization is oriented along the *x* direction corresponding to the direction of initial saturation. The volume of ordered FeRh where the magnetization evolves

with the temperature is in the form of a rectangular wire with a 50x80 nm^2 section and a core-shell structure. The shell takes into account the fact that the magnetic transition at surfaces/interfaces is different from that in the core in which the FM and AFM areas nucleate and coalesce. The shell thickness was taken as the average of the measured widths for a fixed temperature (Figure 4a)), and considered to be uniform over the entire section of the layer in the simulations for simplicity. The thickness of the shell and its magnetization parallel to the *x* direction increase gradually with temperature (6 nm/ 0.8 T, 9 nm / 1 T and 12 nm / 1.19 T for 25°C, 50°C and 60°C respectively). The mean magnetization of the FM domains (0.94 T at 50°C and 1.07 T at 60°C) was extracted from the central portion of Figure 4a) and set parallel to the *x* direction, while the magnetization of AFM areas is considered equal to 0. The period of the FM domains is 100 nm and their lateral size was adjusted accordingly to the experimental phase images (20 nm to 50°C, 40 nm to 60°C). From the micromagnetic simulations of this system, the components of the magnetization and the dipolar field have been extracted to calculate the total magnetic induction. After integrating the components perpendicular to the electron beam along the electron path, the corresponding simulated phase image was calculated using Aharonov-Bohm equations and compared directly to experimental magnetic phase images.

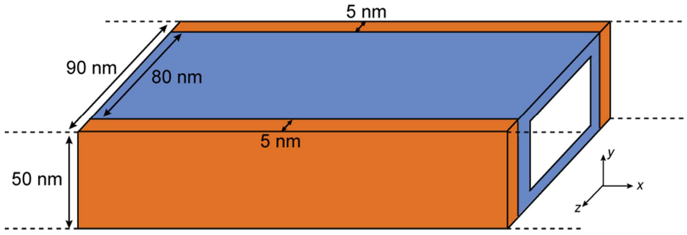

Supplementary Figure 5 | 3D scheme of the system used for the micromagnetic simulations. In orange are given the damaged surfaces kept in the F state. The white and blue parts correspond respectively to the core and shell parts of the ordered FeRh layer where the AF/F transition appears. The shell presents a varying thickness with the temperature.

Supplementary references

1. Harada, K., Tonomura, A., Togawa, Y., Akashi, T. & Matsuda, T. Double-biprism electron interferometry. *Appl. Phys. Lett.* **84,** 3229–3231 (2004).
2. Tonomura, A. in *Progress in Optics* (ed. E. Wolf) **23,** 183–220 (Elsevier, 1986).
3. Tonomura, A. Applications of electron holography. *Rev. Mod. Phys.* **59,** 639–669 (1987).
4. Aharonov, Y. & Bohm, D. Significance of Electromagnetic Potentials in the Quantum Theory. *Phys. Rev.* **115,** 485–491 (1959).
5. Aharonov, Y. & Bohm, D. Further Considerations on Electromagnetic Potentials in the Quantum Theory. *Phys. Rev.* **123,** 1511–1524 (1961).
6. Midgley, P. A. & Dunin-Borkowski, R. E. Electron tomography and holography in materials science. *Nat. Mater.* **8,** 271–280 (2009).
